# Proton pump inhibitors affect capecitabine efficacy in patients with stage II–III colorectal cancer: a multicenter retrospective study

**DOI:** 10.1038/s41598-022-10008-2

**Published:** 2022-04-21

**Authors:** Yoshiko Kitazume, Hitoshi Kawazoe, Ryuji Uozumi, Tomoe Yoshizawa, Hirotoshi Iihara, Hironori Fujii, Masaya Takahashi, Takahiro Arai, Yasushi Murachi, Yumiko Sato, Takahiro Mikami, Koji Hashiguchi, Tomoko Yamazaki, Katsuyuki Takahashi, Yukiyoshi Fujita, Yuki Hosokawa, Issei Morozumi, Masami Tsuchiya, Atsushi Yokoyama, Hironobu Hashimoto, Masakazu Yamaguchi

**Affiliations:** 1 Department of Pharmacy, National Cancer Center Hospital, 5-1-1 Tsukiji, Chuo-ku, Tokyo, 104-0045 Japan; 2 Division of Pharmaceutical Care Sciences, Center for Social Pharmacy and Pharmaceutical Care Sciences, Keio University Faculty of Pharmacy, 1-5-30 Shibakoen, Minato-ku, Tokyo, 105-8512 Japan; 3 Division of Pharmaceutical Care Sciences, Keio University Graduate School of Pharmaceutical Sciences, 1-5-30 Shibakoen, Minato-ku, Tokyo, 105-8512 Japan; 4 Department of Biomedical Statistics and Bioinformatics, Kyoto University Graduate School of Medicine, 54 Kawahara-cho, Shogoin, Sakyo-ku, Kyoto, 606-8507 Japan; 5 Department of Pharmacy, Tochigi Cancer Center, 4-9-13 Yohnan, Utsunomiya, Tochigi, 320-0834 Japan; 6 Department of Pharmacy, Gifu University Hospital, 1-1 Yanagido, Gifu, Gifu, 501-1194 Japan; 7 Department of Pharmacy, Osaka City University Hospital, 1-5-7 Asahi-machi, Abeno-ku, Osaka, 545-8586 Japan; 8 Division of Pharmacy, Gunma Prefectural Cancer Center, 617-1 Takahayashi-nishi-cho, Ota, Gunma 373-0828 Japan; 9 Department of Pharmacy, Independent Administrative Institution Higashiosaka City Medical Center, 3-4-5 Nishiiwata, Higashiosaka, Osaka 578-8588 Japan; 10 Department of Frontier Science for Cancer and Chemotherapy, Osaka University Graduate School of Medicine, 2-2 Yamadaoka, Suita, Osaka 565-0871 Japan; 11 Department of Pharmacy, Nagoya City University West Medical Center, 1-1-1 Hirate-cho, Kita-ku, Nagoya, Aichi 462-8508 Japan; 12 Department of Pharmacy, Miyagi Cancer Center, 47-1 Nodayama, Medeshimashiote, Natori, Miyagi 981-1293 Japan; 13 Department of Pharmacy, Yokohama Minami Kyousai Hospital, 1-21-1 Mutsuurahigashi, Kanazawa-ku, Yokohama, Kanagawa 236-0037 Japan; 14 Department of Pharmacy, Cancer Institute Hospital, Japanese Foundation for Cancer Research, 3-8-31 Ariake, Koto-ku, Tokyo, 135-8550 Japan

**Keywords:** Cancer, Oncology

## Abstract

The association between capecitabine efficacy and proton pump inhibitors (PPIs) is controversial. Here, we determined whether co-administration of PPIs affects the real-world effectiveness of capecitabine. This retrospective observational study included consecutive patients with stage II–III colorectal cancer (CRC) who received adjuvant capecitabine monotherapy or CapeOX (capecitabine and oxaliplatin) between January 2009 and December 2014 at nine participating institutions. The primary endpoint was the difference in relapse-free survival (RFS) between patients who received PPIs and those who did not and was estimated using the Kaplan–Meier method. Overall survival (OS) was the secondary endpoint. Multivariable analysis of RFS and OS was performed using a Cox proportional hazards model, propensity score adjustment, and inverse probability of treatment weighting (IPTW) analyses. Data from 606 patients were evaluated, 54 of whom had received a PPI. PPI-treated patients tended to have poorer RFS and OS than patients treated without PPIs. The hazard ratio for RFS with capecitabine monotherapy was 2.48 (95% confidence interval: 1.22–5.07). These results were consistent with sensitivity analyses performed using propensity score adjustment and IPTW methods. Co-administration of PPIs may reduce the effectiveness of capecitabine and negatively impact patients with stage II–III CRC.

## Introduction

Colorectal cancer (CRC) is the third most common cancer in males and second most common cancer in females, according to the World Health Organization GLOBOCAN database, having caused ~ 861,000 deaths in 2018^1^. In patients with stage II–III CRC, postoperative adjuvant chemotherapy is a standard treatment to reduce the recurrence rate^2–4^, and capecitabine monotherapy or in combination with oxaliplatin is standard adjuvant chemotherapy for patients with early-stage CRC^5,6^. Capecitabine is an orally available prodrug of 5-fluorouracil (5-FU), activated through a three-step enzymatic process, allowing the entry of higher concentrations of 5-FU in tumor tissues^7,8^.

Recently, co-administration of proton pump inhibitors (PPIs) with capecitabine has become controversial^9^, and studies report that concomitant use of PPIs and capecitabine may reduce the efficacy of capecitabine and lead to poor survival outcomes^10–13^. In a *post-hoc* analysis of a phase III clinical trial, concurrent use of PPIs with the CapeOX regimen (capecitabine and oxaliplatin) negatively impacted the outcomes of 545 patients with advanced or metastatic gastroesophageal cancer^11^. One mechanism proposed for the drug–drug interaction between PPIs and capecitabine is reduced dissolution of the capecitabine tablet due to increased gastric pH induced by the concomitant use of PPIs, leading to decreased capecitabine absorption^13^. However, in vitro data do not support the existence of this drug–drug interaction^14^, and few pharmacokinetic studies assessing the interaction between capecitabine and PPIs have been conducted in clinical settings. Sekido et al.^15^ reported that co-administration of rabeprazole, a PPI, did not impact the pharmacokinetics of capecitabine or its metabolites in patients with CRC, suggesting no pharmacokinetic interaction between these two drugs. Therefore, against this background, we hypothesized that co-administration of PPIs does not reduce capecitabine efficacy. However, the prospective study of Sekido et al.^15^ included only 14 patients and involved the use of rabeprazole only. Thus, the existence of a drug–drug interaction between PPIs and capecitabine in clinical practice remains unclear. Additionally, to the best of our knowledge, this potential drug–drug interaction has not been investigated in postoperative patients with stage II–III CRC receiving adjuvant chemotherapy in a large-scale, multicenter study.

CapeOX is “preferred” over capecitabine monotherapy because adding oxaliplatin to capecitabine improved outcomes in stage III colorectal cancer patients^2–4,16^. In addition, the CapeOX regimen is one of the most selected regimens in current clinical practice settings. Therefore, we decided that the drug–drug interaction between PPIs and CapeOX regimen should also be investigated.

This study aimed to clarify whether co-administration of PPIs affects the real-world effectiveness of capecitabine monotherapy and the CapeOX regimen in patients with early-stage CRC.

## Results

### Patient characteristics

**Figure 1:**
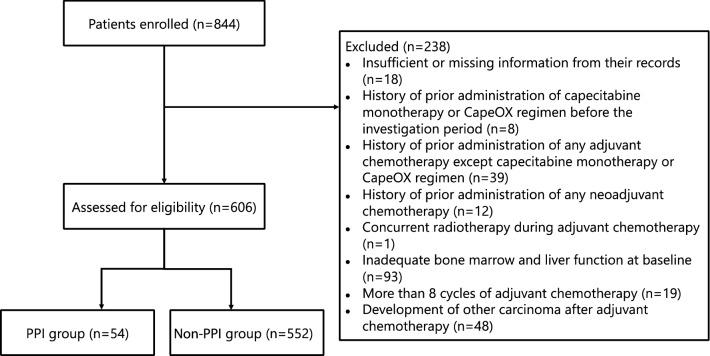
Patient enrollment flowchart. Abbreviations: CapeOX, capecitabine and oxaliplatin; PPI, proton pump inhibitor.

Eight hundred forty-four patients were initially screened, and 238 patients were withdrawn from the analysis based on the exclusion criteria listed in the Methods section. Data of 606 patients were evaluated in this study (Fig. 1). Among these, 54 (8.9%) patients received PPIs, with 29 (53.7%) and 25 (46.3%) receiving capecitabine monotherapy and CapeOX regimen, respectively.

The baseline patient characteristics are listed in Table 1. The median age of the patients was 63 years [interquartile range (IQR): 55–70 years], 328 (54.1%) were males, 171 (28.2%) had right-sided colon cancer, and 533 (88.0%) had stage III CRC. The median duration of concomitant PPI use was 72.8% (IQR: 46.9–100%).

**Table 1:** Baseline patient characteristics.

Characteristic	All (n = 606)	PPI group (n = 54)^a^		Non-PPI group (n = 552)^a^	
		Capecitabine monotherapy (n = 29)	CapeOX (n = 25)	Capecitabine monotherapy (n = 420)	CapeOX (n = 132)
Age, median (IQR), y	63 (55–70)	63 (58–70)	61 (55–66)	64 (57–71)	60 (51–67)
**Sex**					
Male	328 (54.1)	11 (37.9)	12 (48.0)	226 (53.8)	79 (59.8)
Female	278 (45.9)	18 (62.1)	13 (52.0)	194 (46.2)	53 (40.2)
Primary site^a^					
Right-sided colon	171 (28.2)	7 (24.1)^b^	3 (12.0)	123 (29.3)^c^	38 (28.8)
Left-sided colon	215 (35.5)	8 (27.6)	7 (28.0)	161 (38.3)^d^	39 (29.5)^e^
Rectum	220 (36.3)	14 (48.3)^f^	15 (60.0)	136 (32.4)^g^	55 (41.7)
**Stage**					
II	73 (12.0)	6 (20.7)	1 (4.0)	60 (14.3)	6 (4.5)
IIIA	106 (17.5)	6 (20.7)	4 (16.0)	81 (19.3)	15 (11.4)
IIIB	341 (56.3)	14 (48.3)	12 (48.0)	237 (56.4)	78 (59.1)
IIIC	86 (14.2)	3 (10.3)	8 (32.0)	42 (10.0)	33 (25.0)
Co-administered PPIs					
Lansoprazole	30 (55.6)	13 (44.8)	17 (68.0)		
Esomeprazole	10 (18.5)	6 (20.7)	4 (16.0)		
Rabeprazole	10 (18.5)	7 (24.1)	3 (12.0)		
Omeprazole	4 (7.4)	3 (10.3)	1 (4.0)		

^a^Percentages may not add up to 100 because of rounding.

^b^One patient had concurrent sigmoid colon cancer.

^c^Five patients had concurrent gastric cancer (n = 2), both cecum and transverse colon cancers (n = 1) (because cecum cancer was more malignant than transverse colon cancer, the patient was registered as having cecum cancer), and sigmoid colon cancer (n = 2).

^d^Eight patients had concurrent head and neck cancer (n = 1), gastric cancer (n = 3), transverse colon cancer (n = 1), and rectal cancer (n = 3).

^e^Three patients had concurrent gastric cancer (n = 2), and prostate cancer (n = 1).

^f^One patient had concurrent transverse colon cancer.

^g^One patient had concurrent transverse colon cancer.

Abbreviations: CapeOX, capecitabine and oxaliplatin; IQR, interquartile range; PPI, proton pump inhibitor.

### Endpoints

The median duration of follow-up was 6.1 years (95% confidence interval [CI]: 5.9–6.3 years). Overall, there were 125 relapse events and 74 deaths. Among patients who received capecitabine monotherapy, the median relative dose intensity (RDI) of capecitabine was 66.1% (IQR: 50.9–74.1%) and 79.6% (IQR: 64.9–91.2%) in the PPI and non-PPI groups, respectively. Among patients who received the CapeOX regimen, the median RDI of capecitabine was 72.9% (IQR: 63.6–88.2%) and 75.9% (IQR: 62.6–87.7%) in the PPI and non-PPI groups, respectively, and the median RDI of oxaliplatin was 73.3% (IQR: 63.3–88.2%) and 66.6% (IQR: 46.7–79.2%) in the PPI and non-PPI groups, respectively.

**Figure 2:**
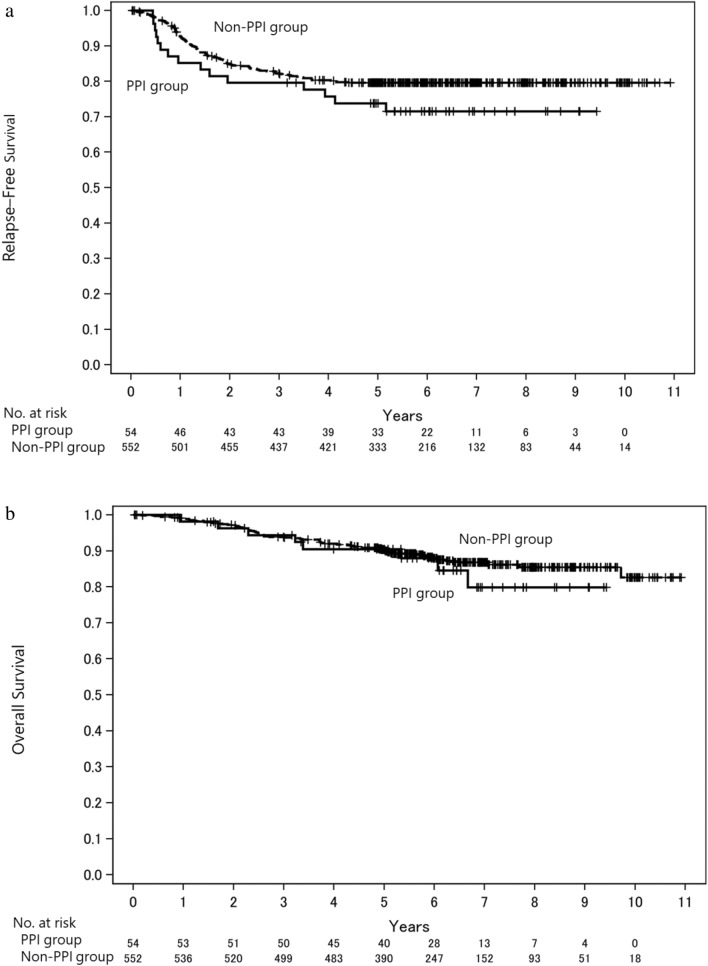
Kaplan–Meier curves for (**a**) relapse-free survival and (**b**) overall survival according to the absence or presence of PPIs in the entire population (capecitabine monotherapy and CapeOX regimen). Abbreviations: PPI, proton pump inhibitor.

As shown in Fig. 2, relapse-free survival (RFS) at 5 years was 73.8% (95% CI: 59.8–83.6%) and 79.6% (95% CI: 75.9–82.8%) in the PPI and non-PPI groups, respectively. Overall survival (OS) at 5 years was 90.5% (95% CI: 78.6–95.9%) and 90.4% (95% CI: 87.6–92.6%) in the PPI and non-PPI groups, respectively. PPI-treated patients showed poorer RFS (hazard ratio [HR], 1.44; 95% CI: 0.84–2.47; *P* = 0.185) and OS (HR, 1.25; 95% CI: 0.60–2.60; *P* = 0.558) than patients treated without PPIs according to univariable analysis.

As shown in Table 2, in the entire population (capecitabine monotherapy and CapeOX regimen), multivariable Cox proportional hazards model, propensity score-adjusted, and inverse probability of treatment weighting (IPTW) analyses revealed that co-administration of PPIs was associated with a poor RFS, although the result was not statistically significant (HR, 1.43; 95% CI: 0.81–2.51, *P* = 0.215; HR, 1.37; 95% CI: 0.79–2.41, *P* = 0.265; and HR, 1.54; 95% CI: 0.85–2.80; *P* = 0.158, respectively). Similar results were obtained for OS using multivariable Cox proportional hazards model, propensity score-adjusted, and IPTW analyses (Table 3). Besides, an increase of RDI was associated with the reduced RFS and OS (HR, 0.93; 95% CI: 0.86–1.01; *P* = 0.088 and HR, 0.88; 95% CI: 0.80–0.97; *P* = 0.011, respectively), and stage III high-risk was associated with increased RFS and OS (HR, 2.33; 95% CI: 1.26–4.30; *P* = 0.007 and HR, 2.78; 95% CI: 1.23–6.29; *P* = 0.014, respectively).

**Table 2:** Multivariable Cox proportional hazards model, propensity score-adjustment, and IPTW analyses of the effect of co-administration of PPIs on relapse-free survival of the entire population (capecitabine monotherapy and CapeOX regimen).

					Multivariable analysis			Adjusted for propensity score			IPTW		
Variables		No.	Event	Censored	HR (95% CI)	*P-value*	Posterior probability	HR (95% CI)	*P-value*	Posterior probability	HR (95% CI)	*P-value*	Posterior probability
PPI	Yes	54	15	39	1.43 (0.81–2.51)	0.215	0.135	1.37 (0.79–2.41)	0.265	0.159	1.54 (0.85–2.80)	0.158	0.077
	No	552	110	442	1			1			1		
Age (10-year intervals)		–	–	–	0.89 (0.75–1.05)	0.178							
Sex	Male	328	76	252	1.40 (0.97–2.01)	0.072							
	Female	278	49	229	1								
Primary site	Right-sided colon	171	39	132	1.13 (0.77–1.67)	0.536							
	Others	435	86	349	1								
Stage	III high-risk	199	67	132	2.33 (1.26–4.30)	0.007							
	III low-risk	334	45	289	0.78 (0.42–1.46)	0.443							
	II	73	13	60	1								
Regimen	CapeOX	157	39	118	0.90 (0.59–1.38)	0.639							
	Capecitabine	449	86	363	1								
RDI (10% intervals)		–	–	–	0.93 (0.86–1.01)	0.088							

Abbreviations: CapeOX, capecitabine and oxaliplatin; CI, confidence interval; HR, hazard ratio; IPTW, inverse probability of treatment weighting; PPI, proton pump inhibitor; RDI, relative dose intensity.

**Table 3:** Multivariable Cox proportional hazards model, propensity score-adjustment, and IPTW analyses of the effect of co-administration of PPIs on overall survival of the entire population (capecitabine monotherapy and CapeOX regimen).

					Multivariable analysis			Adjusted for propensity score			IPTW		
Variables		No.	Event	Censored	HR (95% CI)	*P-value*	Posterior probability	HR (95% CI)	*P-value*	Posterior probability	HR (95% CI)	*P-value*	Posterior probability
PPI	Yes	54	8	46	1.26 (0.58–2.71)	0.560	0.308	1.12 (0.53–2.40)	0.767	0.422	1.21 (0.54–2.73)	0.642	0.347
	No	552	66	486	1			1			1		
Age (10-year intervals)		–	–	–	0.95 (0.76–1.20)	0.683							
Sex	Male	328	48	280	1.57 (0.97–2.56)	0.066							
	Female	278	26	252	1								
Primary site	Right-sided colon	171	26	145	1.27 (0.78–2.08)	0.336							
	Others	435	48	387	1								
Stage	III high-risk	199	47	152	2.78 (1.23–6.29)	0.014							
	III low-risk	334	20	314	0.63 (0.27–1.50)	0.300							
	II	73	7	66	1								
Regimen	CapeOX	157	24	133	0.95 (0.55–1.64)	0.855							
	Capecitabine	449	50	399	1								
RDI (10% intervals)		–	–	–	0.88 (0.80–0.97)	0.011							

Abbreviations: CapeOX, capecitabine and oxaliplatin; CI, confidence interval; HR, hazard ratio; IPTW, inverse probability of treatment weighting; PPI, proton pump inhibitor; RDI, relative dose intensity.

Comparison of capecitabine monotherapy with the CapeOX regimen (Supplementary Figure S1 and S2) showed that the HR for RFS with capecitabine monotherapy was 1.90 (95% CI: 0.95–3.79; *P* = 0.069) according to univariable analysis, and RFS at 5 years was 72.2% (95% CI: 52.1–85.0%) and 81.4% (95% CI: 77.3–84.8%) in the PPI and non-PPI groups, respectively (Supplementary Figure S1a). In contrast, the HR for RFS with the CapeOX regimen was 0.87 (95% CI: 0.36–2.07; *P* = 0.751) according to univariable analysis (Supplementary Figure S2a). Sensitivity analyses using the multivariable Cox proportional hazards model, propensity score-adjusted, and IPTW analyses for RFS and OS demonstrated consistent results (Tables S1, S2, S3, and S4). In capecitabine monotherapy, the HRs for RFS calculated by multivariable Cox proportional hazards model, propensity score-adjusted, and IPTW analyses were 2.48 (95% CI: 1.22–5.07; *P* = 0.013), 2.18 (95% CI: 1.08–4.41; *P* = 0.030), and 1.99 (95% CI: 0.87–4.59; *P* = 0.104), respectively.

In the entire population (capecitabine monotherapy and CapeOX regimen), the Bayesian posterior probability that the HRs for the RFS of the PPI group relative to those of the non-PPI group would be < 1.00 ranged from 7.7% to 15.9% (Table 2). In the capecitabine monotherapy population, the Bayesian posterior probability that the HRs for the RFS of the PPI group relative to those of the non-PPI group would be < 1.00 ranged from 1.4% to 4.7% (Supplementary Table S1).

## Discussion

This study showed that co-administration of PPIs might reduce the effectiveness of capecitabine and negatively impact outcomes. The two propensity score-adjusted analyses using a logistic regression model including independent variables such as RDI and stage III high-risk showed a similar tendency. We found that patients treated with PPIs had reduced RFS (increased risk: 37–54%) and OS (increased risk: 12–26%) relative to patients treated without PPIs. These results do not support our original hypothesis that co-administration of PPIs does not reduce the efficacy of capecitabine. To the best of our knowledge, this is the first multicenter study using real-world data to investigate whether co-administration of PPIs affects the efficacy of capecitabine monotherapy and CapeOX regimen in patients with stage II–III CRC.

Previous studies have reported that PPIs combined with capecitabine monotherapy or CapeOX regimen reduce the effectiveness of capecitabine and negatively impact survival in patients with local and advanced CRC or gastroesophageal cancer^10–13^. Notably, the results of a *post-hoc* analysis of the TRIO-013/LOGiC phase III trial (n = 545) impacted clinical practice because data quality was robust, and the sample size was relatively large^11^. The results of the present study are consistent with those findings; however, evidence of a drug–drug interaction between PPIs and capecitabine is inconsistent among data obtained in the clinic, in vitro*,* and from pharmacokinetic studies^14,15^. Importantly, Sekido et al.^15^ reported no pharmacokinetic interaction between capecitabine and rabeprazole, and their data do not support the results of the present study. The mechanism underlying the drug–drug interaction between PPIs and capecitabine has not been fully clarified^14,15^. Direct promotion of CRC by PPIs is one possible mechanism. Some studies have shown that PPI use is significantly associated with CRC risk, whereas several meta-analyses have shown no association between PPI use and CRC risk^17–20^. The effects of PPIs on CRC remain controversial; however, Wong et al.^13^ reported that PPIs had no significant effect on the 3-year RFS of 5-FU-, leucovorin-, and oxaliplatin (FOLFOX)-treated patients with early-stage CRC. Furthermore, Kim et al.^21^ showed that concomitant use of PPI was associated with better progression-free survival and OS in 5-FU-, leucovorin-, and irinotecan-treated patients with advanced CRC compared with that in modified capecitabine plus irinotecan-treated patients with advanced CRC. This suggests that the effect of PPIs on the RFS of patients who receive capecitabine might be the result of a drug–drug interaction with capecitabine rather than an effect on CRC itself. Surprisingly, it was recently highlighted that the gut microbiome controls the response to chemotherapy, especially immunotherapy^9,22^, and preclinical data show that bacteria can modulate the efficacy of some chemotherapeutic agents, although this has not yet been reported for capecitabine^9,23^. Conversely, capecitabine is metabolized to its active form, 5-FU, by thymidine phosphorylase, which is enriched in tumors^7,8^, and pharmacokinetic data indicate that PPIs might affect thymidine phosphorylase in tumor cells^15^. Further fundamental in vivo and in vitro studies are warranted to clarify these mechanisms.

In data presented in Fig. 1, we excluded 93 patients with inadequate bone marrow and liver function at baseline because we wanted to evaluate the patients according to the Japanese Society for Cancer of the Colon and Rectum (JSCCR) guidelines, which have been used as a reference for treating colorectal cancer in actual clinical practice settings in Japan. Between January 2009 and December 2014, although three sets of guidelines were published (JSCCR guidelines 2009 was not translated in English)^24,25^, the general principles underlying the indications for systemic chemotherapy were not changed, except those for the white blood cell count; bone marrow function was maintained at peripheral white blood cell count > 3500 or 4000 cells/mm^3^ and platelet count > 100,000 cells/mm^3^, and liver function was maintained at total bilirubin < 2.0 mg/dL and aspartate transaminase/alanine transaminase < 100 IU/L. The JSCCR guidelines 2009 recommended that postoperative adjuvant chemotherapy be initiated at ~ 4 to 8 weeks after surgery, so postoperative adjuvant chemotherapy was possibly introduced for patients whose bone marrow or liver function had not fully recovered. In addition, in clinical practice, postoperative adjuvant chemotherapy is conducted after obtaining informed consent from the patient regarding various factors, such as prognosis, complications, and expected quality of life after treatment. Thus, postoperative adjuvant chemotherapy is considered to have been performed even if bone marrow or liver function was insufficient. In this study, we included the patients who met the criteria (JCSSR guidelines) for indication to fairly compare the PPI and non-PPI groups.

In the present study, the survival curve for patients who received the CapeOX regimen was the inverse of that for patients who received capecitabine monotherapy (Supplementary Figure S1 and S2). This suggested that if PPIs suppress capecitabine effectiveness, combination treatment with oxaliplatin might counteract this attenuating effect. Since the efficacy of oxaliplatin combination therapy (i.e., CapeOX regimen, FOLFOX regimen) is superior to fluoropyrimidine monotherapies [i.e., 5-FU and leucovorin, capecitabine, tegafur gimeracil oteracil potassium (S-1)], CapeOX regimen is “preferred” over capecitabine monotherapy^2–4,16^. Oncologists and clinicians should consider each patient’s risk of recurrence and expected efficacy, adverse events, treatment cost, and the number of hospital visits while selecting an appropriate regimen. In addition, it is recommended that the treatment regimen should be selected based on a comprehensive judgment that includes the patient’s systemic condition and willingness to receive treatment. Based on the results of the present study, if a patient has a reason to continue using PPIs during postoperative adjuvant chemotherapy, CapeOX regimen may be an option for them.

From Tables 2 and 3, an increase of RDI was associated with the reduced RFS and OS (HR, 0.93; 95% CI: 0.86–1.01; *P* = 0.088 and HR, 0.88; 95% CI: 0.80–0.97; *P* = 0.011, respectively). This result suggests that increased RDI is identified as one of the good prognostic factors for postoperative adjuvant chemotherapy, and it is important to maintain high dose intensity. In order to keep high dose intensity, pharmacists should work to improve patients’ medication adherence and detect adverse events earlier so that patients can continue their treatment.

Geriatric populations often have issues with polypharmacy. PPIs are frequently prescribed, and 25‒70% of PPIs can potentially be prescribed inappropriately^26^. The findings based on the present and previous studies indicate that pharmacists should intervene and encourage oncologists and clinicians to replace PPIs with alternative gastric acid suppressants, such as Maalox^27^ or mucosa-protective agents on an as-needed basis. Additionally, PPIs are prescribed to treat peptic ulcers, gastroesophageal reflux disease, Zollinger–Ellison syndrome, ulcers induced by non-steroidal anti-inflammatory drugs, and eradication of *Helicobacter pylori*. It is essential to determine the initial indication for a PPI and resolve the issue without PPI administration. For example, if a patient is diagnosed with peptic ulcer, pharmaceutical interventions should be provided along with support to reduce stress, and a review of the eating habits of the patient should be performed.

The present study has several strengths. First, it was a multicenter study with nine participating institutions, including cancer centers, university hospitals, and community hospitals in Japan. Therefore, we believe that our data may be generalizable to similar populations in a clinical setting. Second, the sample size was relatively large, similar to phase III clinical trials, although the number of PPI-treated patients was low. Third, we focused on adjuvant chemotherapy for postoperative patients with stage II–III CRC. Fourth, there was a long follow-up period. Fifth, we performed sensitivity analyses using multivariable Cox proportional hazards model, propensity score-adjusted, and IPTW methods, providing consistent results. These analyses increased the robustness of the results and are a novel feature of our study.

The present study did have several limitations. First, the number of PPI-treated patients was only 29 on capecitabine monotherapy and only 25 on CapeOX regimen, and the number of events in PPI-treated patients (15 relapse events and 8 deaths) was also small. Second, we did not include comorbidity data as independent variable in the multivariable model, although PPI-treated patients may be more comorbid than patients treated without PPIs. We did not include the comorbidity data for two reasons. The first reason is that we did not collect comorbidity data because we considered that information on comorbidities was not available in every institution because of the retrospective nature of the study. The second reason is that we considered that patients with serious complications would not undergo surgery and postoperative adjuvant chemotherapy. Third, it was a retrospective, observational study rather than a prospective study, and therefore, the occurrence of information bias cannot be excluded. Thus, methodologically, the two groups were not matched, and the sample size was unequal. For this reason, we used multivariable analyses to reduce the effect of potential confounding factors associated with observational studies and clinical differences in patient characteristics. Nevertheless, unmeasured confounders could not be controlled during multivariable analyses. Fourth, since this was a retrospective observational study, we could not identify the reasons for taking PPIs. Fifth, in cancer patients, a PPI is usually considered to control adverse events (e.g., nausea), which is directly related to compliance with oral medications. However, compliance-related information was also not available because this was a retrospective observational study. Sixth, co-administration of PPIs was determined based on prescription histories; therefore, it was unclear whether the patients took PPIs during capecitabine administration. Seventh, the present study did not evaluate the pharmacokinetics of capecitabine; however, in vitro data demonstrate that capecitabine tablets are readily dissolved at pH > 4 (i.e., pH 4.5 or 6.8), with > 85% of the tablet dissolved after 30 min^14^. Additionally, a previous pharmacokinetic study indicated a lack of association between the concurrent use of PPIs and the blood concentration of capecitabine^15^. Finally, these results do not clarify the potential mechanism underlying the reduced effectiveness of capecitabin and the poorer survival outcomes in PPI-treated patients. Overall, the findings of this study should be confirmed in a prospective study with adequate sample size and utilizing a pharmacokinetic approach.

In conclusion, the findings of this study suggest that concomitant use of PPIs may reduce the real-world effectiveness of capecitabine and negatively impact the survival outcomes for patients with stage II–III CRC, although the limitations must be considered. In order to evaluate the effect of the concomitant use of capecitabine and PPI on RFS and OS in patients with CRC, it is necessary to include patients treated with other oral anticancer drugs (e.g., S-1), rather than limiting future studies to patients treated with capecitabine. Sekido et al.^15^ showed no pharmacokinetic interaction between capecitabine and only rabeprazole among several types of PPIs, which provides very limited information. The best way to elucidate the pharmacokinetic interaction between capecitabine and PPIs is to conduct randomized studies with a homogenous design, including pharmacokinetic analysis regardless of the type of PPIs used and the type of chemotherapy. Our data provide preliminary evidence for an association between capecitabine efficacy and PPI use in Japanese patients with stage II–III CRC. These findings may be translatable to other Asian populations and highlight the need for additional research in this field.

## Methods

### Patients

Nine institutions participated in this retrospective observational study, including cancer centers, university hospitals, and community hospitals in Japan, and all patients enrolled in this study were of Asian heritage. Data were collected from the medical records of each institution. The inclusion criteria were as follows: 1) consecutive patients aged ≥ 20 years with pathologically diagnosed stage II–III CRC and who had undergone curative surgery, and 2) patients who had received at least one course of adjuvant capecitabine monotherapy (2500 mg/m^2^ days 1–14 every 3 weeks) or CapeOX regimen (capecitabine 2000 mg/m^2^ days 1–14 and oxaliplatin 130 mg/m^2^ day 1 every 3 weeks) between January 2009 and December 2014. The clinicopathologic findings were reclassified according to the TNM Classification of Malignant Tumors, 8^th^ edition of the Union for International Cancer Control^28^. The treatment schedule and follow-up duration were modified at the clinician’s discretion according to the toxicity profile of each patient.

Patients were excluded from the study based on the following criteria: 1) refused use of medical records; 2) insufficient or missing information from medical records; 3) history of prior administration of capecitabine monotherapy or CapeOX regimen before the investigation period; 4) history of prior administration of any adjuvant chemotherapy, except for capecitabine monotherapy or CapeOX regimen; 5) history of prior administration of any neoadjuvant chemotherapy; 6) concurrent radiotherapy during adjuvant chemotherapy; 7) inadequate bone marrow and liver function at baseline [neutrophil count < 1500 cells/mm^3^ or white blood cell count < 3000 cells/mm^3^; hemoglobin < 9.0 g/dL; platelet count < 75,000 cells/mm^3^; total bilirubin > 2.25 mg/dL; aspartate transaminase > 60 U/L, alanine transaminase > 84 U/L for males and > 46 U/L for females, and creatinine clearance ≤ 51 mL/min as calculated by the Cockcroft–Gault equation]; 8) more than eight cycles of adjuvant chemotherapy; and 9) development of other carcinomas after adjuvant chemotherapy.

### Data collection

Patient records were de-identified and analyzed anonymously. Baseline data (i.e., those collected at the time of treatment initiation) on age, sex, body surface area, cancer stage, primary tumor site, chemotherapy regimen and dose, concomitant PPI use, laboratory data before chemotherapy, and date of recurrence and/or death at the time of adjuvant chemotherapy initiation were collected. The primary site included the right-sided colon (the cecum, ascending colon, and transverse colon), left-sided colon (the descending colon, sigmoid, and rectosigmoid junction), and rectum. Concomitant use of PPI was defined by ≥ 20% overlap of PPI administration during the period of capecitabine administration per a previous study^11^. The end of the follow-up period was December 31, 2019.

RDI of capecitabine or oxaliplatin was defined as the percentage of actual dose intensity per scheduled dose intensity of eight courses. The RDI was calculated as follows:

Actual dose intensity (mg/m^2^/week) = actual total dose (mg) /BSA (m^2^) /actual duration of therapy (week).

Scheduled dose intensity of Capecitabine (mg/m^2^/week) = [2,500 (mg/m^2^/day on capecitabine monotherapy) or 2000 (mg/m^2^/day on CapeOX regimen)] × 14 (day) /3 (week).

Scheduled dose intensity of oxaliplatin (mg/m^2^/week) = 130 (mg/m^2^) /3 (week).

RDI (%) = (actual dose intensity /scheduled dose intensity) × 100.

RFS was defined as the interval from the date of treatment initiation to the date of radiographic recurrence or death from any cause. OS was defined as the interval from the date of treatment initiation to the date of death from any cause. Patients without documented radiographic recurrence or who were still alive were censored on the date of the last follow-up.

### Statistical analysis

The primary and secondary endpoints were RFS and OS, respectively, and estimated using the Kaplan–Meier method according to the drug exposure between patients treated with or without co-administration of PPIs, with CIs calculated using the complementary log–log transformation and Greenwood’s formula. The follow-up period was calculated using reverse Kaplan–Meier estimates^29^. Subsequently, a multivariable Cox proportional hazards model was used to compare differences between the two groups. Potential explanatory variables concerning patient background, including concomitant use of PPI (yes vs. no), age (10-year intervals), sex (male vs. female), primary site [right-sided colon vs. others (left-sided colon and/or rectal)], cancer stage [III high-risk (T4, N2, or both cancers) vs. III low-risk (T1, T2, or T3 and N1 cancers) vs. II], chemotherapy regimen (CapeOX vs. capecitabine), and RDI (10% intervals) were included as independent variables in the multivariable model^4,30–33^. To account for indication bias due to the lack of randomization, two propensity score-adjusted analyses were performed: 1) a multivariable model including propensity score as an additional covariate and 2) an IPTW method^34,35^. The propensity score of co-administration of PPIs was estimated for each patient using a logistic regression model^36^, which included the above-mentioned independent variables. HRs and 95% CIs were calculated. According to the recommendation of the American Statistical Association^37,38^, a *P* < 0.05 should be avoided when interpreting *P*-values; therefore, we interpreted the results based on point estimates with their CIs. Furthermore, to supplement conventional CIs, we performed a *posthoc* analysis using the Cox model re-expressed in a Bayesian statistical framework. We computed the Bayesian posterior probability^39^ of HR < 1 based on a non-informative prior distribution as a reference to evaluate the hypotheses concerning the direction and magnitude of the unknown HR via the Cox model. All statistical analyses were performed using SAS (v.9.4) and JMP (v.15.0.0; SAS Institute, Cary, NC, USA).

## Ethics statement

The study protocol was approved by the National Cancer Center Institutional Review Board (approval number: 2019–294), ethics committee of the Tochigi Cancer Center (approval number: 20-A001), medical review board of Gifu University Graduate School of Medicine (approval number: 2020–069), ethical committee of Osaka City University Graduate School of Medicine (approval number: 2020–042), ethics committee of the Gunma Prefectural Cancer Center (approval number: 405–02,012), ethical review board of Osaka University Hospital (approval number: 20008), Nagoya City University East/West medical center Institutional Review Board (approval number: 20–04-423–03), ethics review committee of the Miyagi Cancer Center (approval number: 2020–003), and ethics committee of the Yokohama Minami Kyosai Hospital (approval number: 1–20-4–1) in Japan. This study was conducted per the Declaration of Helsinki and the Ethical Guidelines for Medical and Health Research involving Human Subjects by the Ministry of Education, Culture, Sports, Science, and Technology and the Ministry of Health, Labour, and Welfare of Japan. Informed consent was waived by the ethics committee of National Cancer Center, Tochigi Cancer Center, Gifu University Graduate School of Medicine, Osaka City University Graduate School of Medicine, Gunma Prefectural Cancer Center, Osaka University Hospital, Nagoya City University East/West medical center, Miyagi Cancer Center, and Yokohama Minami Kyosai Hospital considering retrospective nature of the study.

## Supplementary Information

Supplementary Information.

## Data Availability

The data supporting the findings of this study are available on request from the corresponding authors (H.K. or H.H.) after approval from the ethics committees. The data are not publicly available since they contain information that could compromise the patients’ privacy.
