# Supplementary material for: Proton pump inhibitors affect capecitabine efficacy in patients with stage II–III colorectal cancer: a multicenter retrospective study
Source: Sci Rep. 2022 Apr 21;12:6561. doi: 10.1038/s41598-022-10008-2 (PMC9023444; doi:10.1038/s41598-022-10008-2)
Supplement: Supplementary file 1 — Supplementary Information. [file 41598_2022_10008_MOESM1_ESM.docx]

**Proton Pump Inhibitors Affect Capecitabine Efficacy in Patients with Stage II–III Colorectal Cancer: A Multicenter Retrospective Study**

Yoshiko Kitazume,^1^ Hitoshi Kawazoe,^2,3✉^ Ryuji Uozumi,^4^ Tomoe Yoshizawa,^5^ Hirotoshi Iihara,^6^ Hironori Fujii,^6^ Masaya Takahashi,^7^ Takahiro Arai,^8^ Yasushi Murachi,^9,10^ Yumiko Sato,^11^ Takahiro Mikami,^12^ Koji Hashiguchi,^13^ Tomoko Yamazaki,^5^ Katsuyuki Takahashi,^7^ Yukiyoshi Fujita,^8^ Yuki Hosokawa,^9^ Issei Morozumi,^11^ Masami Tsuchiya,^12^ Atsushi Yokoyama,^13^ Hironobu Hashimoto,^1✉^ Masakazu Yamaguchi^14^

^1^ Department of Pharmacy, National Cancer Center Hospital, 5-1-1 Tsukiji, Chuo-ku, Tokyo 104-0045, Japan

^2^ Division of Pharmaceutical Care Sciences, Center for Social Pharmacy and Pharmaceutical Care Sciences, Keio University Faculty of Pharmacy, 1-5-30 Shibakoen, Minato-ku, Tokyo 105-8512, Japan

^3^ Division of Pharmaceutical Care Sciences, Keio University Graduate School of Pharmaceutical Sciences, 1-5-30 Shibakoen, Minato-ku, Tokyo 105-8512, Japan

^4^ Department of Biomedical Statistics and Bioinformatics, Kyoto University Graduate School of Medicine, 54 Kawahara-cho, Shogoin, Sakyo-ku, Kyoto 606-8507, Japan

^5^ Department of Pharmacy, Tochigi Cancer Center, 4-9-13 Yohnan, Utsunomiya, Tochigi 320-0834, Japan

^6^ Department of Pharmacy, Gifu University Hospital, 1-1 Yanagido, Gifu, Gifu 501-1194, Japan.

^7^ Department of Pharmacy, Osaka City University Hospital, 1-5-7 Asahi-machi, Abeno-ku, Osaka 545-8586, Japan

^8^ Division of Pharmacy, Gunma Prefectural Cancer Center, 617-1 Takahayashi-nishi-cho, Ota, Gunma 373-0828, Japan

^9^ Department of Pharmacy, Independent Administrative Institution Higashiosaka City Medical Center, 3-4-5 Nishiiwata, Higashiosaka, Osaka 578-8588, Japan

^10^ Department of Frontier Science for Cancer and Chemotherapy, Osaka University Graduate School of Medicine, 2-2 Yamadaoka, Suita, Osaka 565-0871, Japan

^11^ Department of Pharmacy, Nagoya City University West Medical Center, 1-1-1 Hirate-cho, Kita-ku, Nagoya, Aichi 462-8508, Japan

^12^ Department of Pharmacy, Miyagi Cancer Center, 47-1 Nodayama, Medeshimashiote, Natori, Miyagi 981-1293, Japan

^13^ Department of Pharmacy, Yokohama Minami Kyousai Hospital, 1-21-1 Mutsuurahigashi, Kanazawa-ku, Yokohama, Kanagawa 236-0037, Japan

^14^ Department of Pharmacy, Cancer Institute Hospital, Japanese Foundation for Cancer Research, 3-8-31 Ariake, Koto-ku, Tokyo 135-8550, Japan

^✉^**Corresponding Author:**

Hitoshi Kawazoe, Ph.D.

Division of Pharmaceutical Care Sciences, Keio University Graduate School of Pharmaceutical Sciences, 1-5-30 Shibakoen, Minato-ku, Tokyo 105-8512, Japan.

Tel: +81-3-5400-2639

Fax: +81-3-5400-2651

E-mail: kawazoe-ht@keio.jp

Hironobu Hashimoto, Ph.D.

Department of Pharmacy, National Cancer Center Hospital, 5-1-1 Tsukiji, Chuo-ku, Tokyo 104-0045, Japan.

Tel: +81-3-3542-2511

Fax: +81-3-3542-0213

E-mail: hhashimo@ncc.go.jp

**Supplementary Table S1. Multivariable Cox proportional hazards model, propensity score-adjustment, and IPTW analyses of the effect of co-administration of PPIs on relapse-free survival with capecitabine monotherapy.**

|  |  |  |  |  | Multivariable analysis | | |  | Adjusted for propensity score | | |  | IPTW | | |
| --- | --- | --- | --- | --- | --- | --- | --- | --- | --- | --- | --- | --- | --- | --- | --- |
| Variables | | No. | Event | Censored | HR (95% CI) | *P*-value | Posterior probability |  | HR (95% CI) | *P*-value | Posterior probability |  | HR (95% CI) | *P*-value | Posterior probability |
| PPI | Yes | 29 | 9 | 20 | 2.48 (1.22–5.07) | 0.013 | 0.014 |  | 2.18 (1.08–4.41) | 0.030 | 0.031 |  | 1.99 (0.87–4.59) | 0.104 | 0.047 |
|  | No | 420 | 77 | 343 | 1 |  |  |  | 1 |  |  |  | 1 |  |  |
| Age (10-year intervals) | | – | – | – | 0.88 (0.72–1.08) | 0.226 |  |  |  |  |  |  |  |  |  |
| Sex | Male | 237 | 50 | 187 | 1.44 (0.93–2.22) | 0.104 |  |  |  |  |  |  |  |  |  |
|  | Female | 212 | 36 | 176 | 1 |  |  |  |  |  |  |  |  |  |  |
| Primary site | Right-sided colon | 130 | 27 | 103 | 1.05 (0.65–1.68) | 0.855 |  |  |  |  |  |  |  |  |  |
|  | Others | 319 | 59 | 260 | 1 |  |  |  |  |  |  |  |  |  |  |
| Stage | III high-risk | 115 | 39 | 76 | 2.75 (1.39–5.43) | 0.004 |  |  |  |  |  |  |  |  |  |
|  | III low-risk | 268 | 36 | 232 | 0.84 (0.42–1.65) | 0.606 |  |  |  |  |  |  |  |  |  |
|  | II | 66 | 11 | 55 | 1 |  |  |  |  |  |  |  |  |  |  |
| RDI (10% intervals) | | – | – | – | 0.96 (0.87–1.06) | 0.400 |  |  |  |  |  |  |  |  |  |

Abbreviations: CI, confidence interval; HR, hazard ratio; IPTW, inverse probability of treatment weighting; PPI, proton pump inhibitor; RDI, RDI, relative dose intensity.

**Supplementary Table S2. Multivariable Cox proportional hazards model, propensity score-adjustment, and IPTW analyses of the effect of co-administration of PPIs on overall survival with capecitabine monotherapy.**

|  |  |  |  |  | Multivariable analysis | | |  | Adjusted for propensity score | | |  | IPTW | | |
| --- | --- | --- | --- | --- | --- | --- | --- | --- | --- | --- | --- | --- | --- | --- | --- |
| Variables | | No. | Event | Censored | HR (95% CI) | *P*-value | Posterior probability |  | HR (95% CI) | *P*-value | Posterior probability |  | HR (95% CI) | *P*-value | Posterior probability |
| PPI | Yes | 29 | 5 | 24 | 2.58 (0.99–6.74) | 0.052 | 0.055 |  | 2.20 (0.86–5.61) | 0.101 | 0.091 |  | 1.95 (0.67–5.71) | 0.222 | 0.113 |
|  | No | 420 | 45 | 375 | 1 |  |  |  | 1 |  |  |  | 1 |  |  |
| Age (10-year intervals) | | – | – | – | 1.07 (0.80–1.44) | 0.635 |  |  |  |  |  |  |  |  |  |
| Sex | Male | 237 | 32 | 205 | 1.71 (0.95–3.08) | 0.072 |  |  |  |  |  |  |  |  |  |
|  | Female | 212 | 18 | 194 | 1 |  |  |  |  |  |  |  |  |  |  |
| Primary site | Right-sided colon | 130 | 16 | 114 | 0.98 (0.53–1.82) | 0.948 |  |  |  |  |  |  |  |  |  |
|  | Others | 319 | 34 | 285 | 1 |  |  |  |  |  |  |  |  |  |  |
| Stage | III high-risk | 115 | 27 | 88 | 2.54 (1.09–5.92) | 0.031 |  |  |  |  |  |  |  |  |  |
|  | III low-risk | 268 | 16 | 252 | 0.54 (0.22–1.31) | 0.173 |  |  |  |  |  |  |  |  |  |
|  | II | 66 | 7 | 59 | 1 |  |  |  |  |  |  |  |  |  |  |
| RDI (10% intervals) | | – | – | – | 0.95 (0.84–1.08) | 0.430 |  |  |  |  |  |  |  |  |  |

Abbreviations: CI, confidence interval; HR, hazard ratio; IPTW, inverse probability of treatment weighting; PPI, proton pump inhibitor; RDI, RDI, relative dose intensity.

**Supplementary Table S3. Multivariable Cox proportional hazards model, propensity score-adjustment, and IPTW analyses of the effect of co-administration of PPIs on relapse-free survival with the CapeOX regimen.**

|  |  |  |  |  | Multivariable analysis | | |  | Adjusted for propensity score | | |  | IPTW | | |
| --- | --- | --- | --- | --- | --- | --- | --- | --- | --- | --- | --- | --- | --- | --- | --- |
| Variables | | No. | Event | Censored | HR (95% CI) | *P*-value | Posterior probability |  | HR (95% CI) | *P*-value | Posterior probability |  | HR (95% CI) | *P*-value | Posterior probability |
| PPI | Yes | 25 | 6 | 19 | 0.82 (0.33–2.00) | 0.658 | 0.711 |  | 0.83 (0.34–2.00) | 0.673 | 0.701 |  | 1.22 (0.50–3.01) | 0.662 | 0.334 |
|  | No | 132 | 33 | 99 | 1 |  |  |  | 1 |  |  |  | 1 |  |  |
| Primary site | Right-sided colon | 41 | 12 | 29 | 1.16 (0.56–2.37) | 0.690 |  |  |  |  |  |  |  |  |  |
|  | Others | 116 | 27 | 89 | 1 |  |  |  |  |  |  |  |  |  |  |
| Stage | III high-risk | 84 | 28 | 56 | 1.05 (0.24–4.57) | 0.944 |  |  |  |  |  |  |  |  |  |
|  | III low-risk | 66 | 9 | 57 | 0.39 (0.08–1.88) | 0.242 |  |  |  |  |  |  |  |  |  |
|  | II | 7 | 2 | 5 | 1 |  |  |  |  |  |  |  |  |  |  |
| RDI (10% intervals) | | – | – | – | 0.89 (0.78–1.02) | 0.089 |  |  |  |  |  |  |  |  |  |

Abbreviations: CI, confidence interval; HR, hazard ratio; IPTW, inverse probability of treatment weighting; PPI, proton pump inhibitor; RDI, RDI, relative dose intensity.

**Supplementary Table S4. Multivariable Cox proportional hazards model, propensity score-adjustment, and IPTW analyses of the effect of co-administration of PPIs on overall survival with the CapeOX regimen.**

|  |  |  |  |  | Multivariable analysis | | |  | Adjusted for propensity score | | |  | IPTW | | |
| --- | --- | --- | --- | --- | --- | --- | --- | --- | --- | --- | --- | --- | --- | --- | --- |
| Variables | | No. | Event | Censored | HR (95% CI) | *P*-value | Posterior probability |  | HR (95% CI) | *P*-value | Posterior probability |  | HR (95% CI) | *P*-value | Posterior probability |
| PPI | Yes | 25 | 3 | 22 | 0.73 (0.21–2.54) | 0.621 | 0.734 |  | 0.69 (0.20–2.34) | 0.551 | 0.780 |  | 0.49 (0.15–1.60) | 0.237 | 0.901 |
|  | No | 132 | 21 | 111 | 1 |  |  |  | 1 |  |  |  | 1 |  |  |
| Primary site | Right-sided colon | 41 | 10 | 31 | 1.85 (0.78–4.37) | 0.160 |  |  |  |  |  |  |  |  |  |
|  | Others | 116 | 14 | 102 | 1 |  |  |  |  |  |  |  |  |  |  |
| Stage | III high-risk | 84 | 20 | 64 | – | 0.991 |  |  |  |  |  |  |  |  |  |
|  | III low-risk | 66 | 4 | 62 | – | 0.992 |  |  |  |  |  |  |  |  |  |
|  | II | 7 | 0 | 7 | 1 |  |  |  |  |  |  |  |  |  |  |
| RDI (10% intervals) | | – | – | – | 0.82 (0.71–0.95) | 0.008 |  |  |  |  |  |  |  |  |  |

Abbreviations: CI, confidence interval; HR, hazard ratio; IPTW, inverse probability of treatment weighting; PPI, proton pump inhibitor; RDI, RDI, relative dose intensity.

**a**


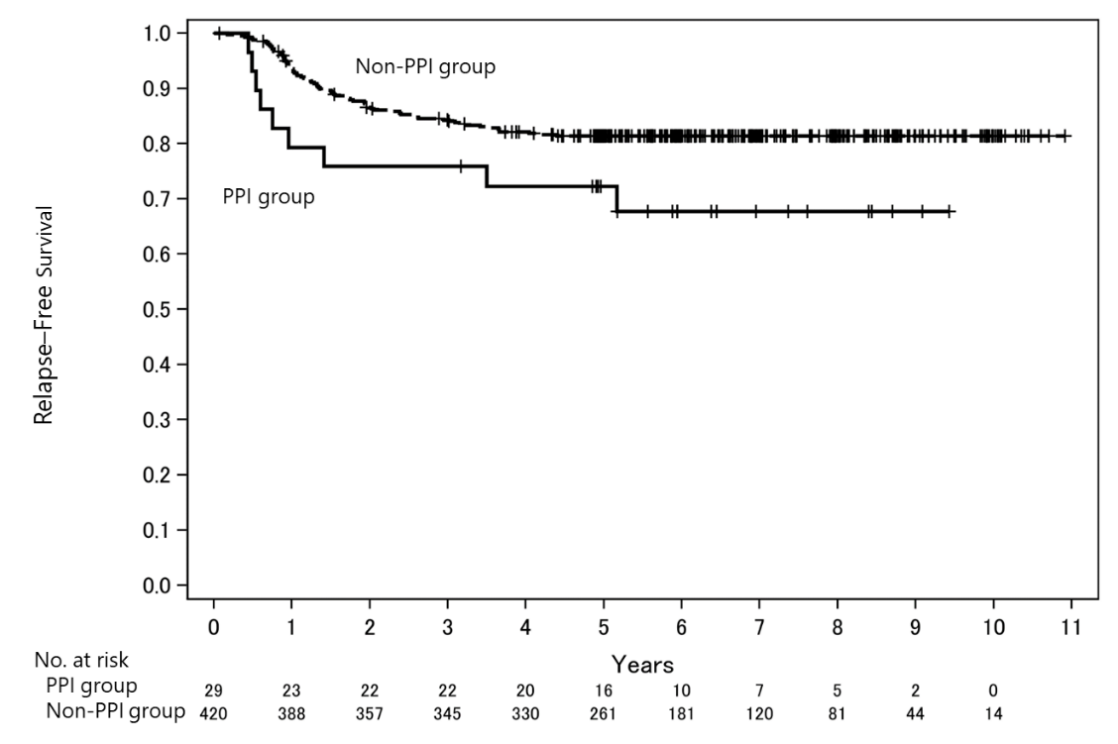


**b**


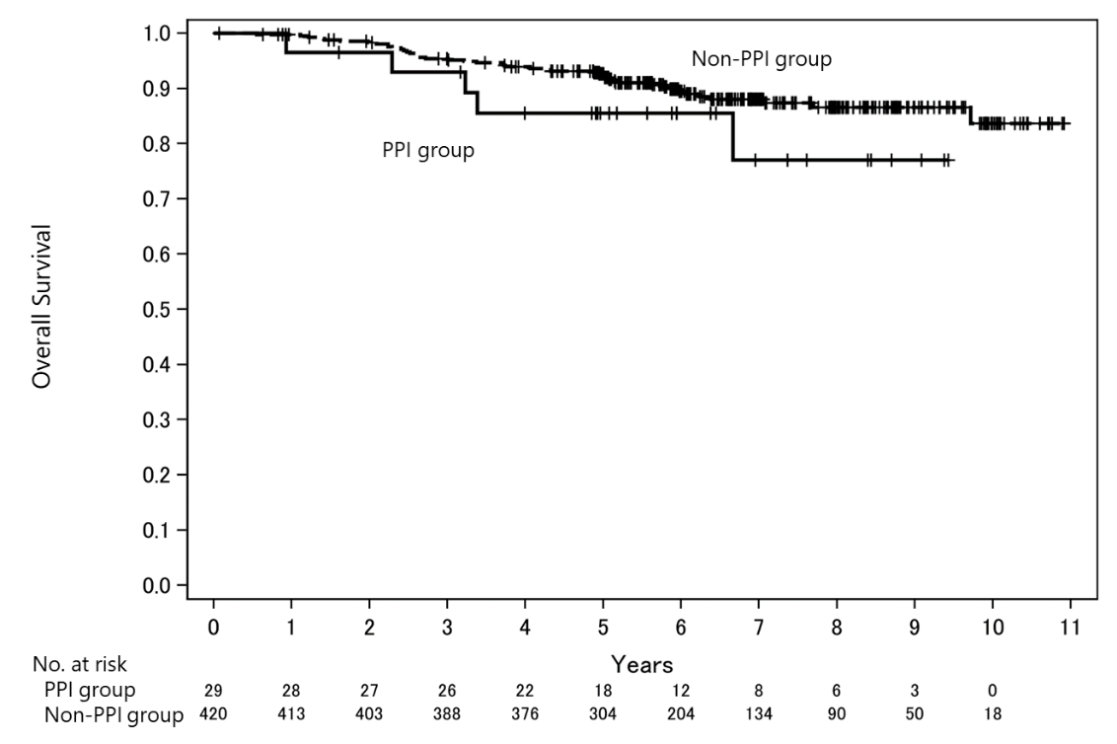


**Supplementary Figure S1.** Kaplan–Meier curves for (**a**) relapse-free survival and (**b**) overall survival according to the absence or presence of PPIs for capecitabine monotherapy.

Abbreviations: PPI, proton pump inhibitor.

**a**


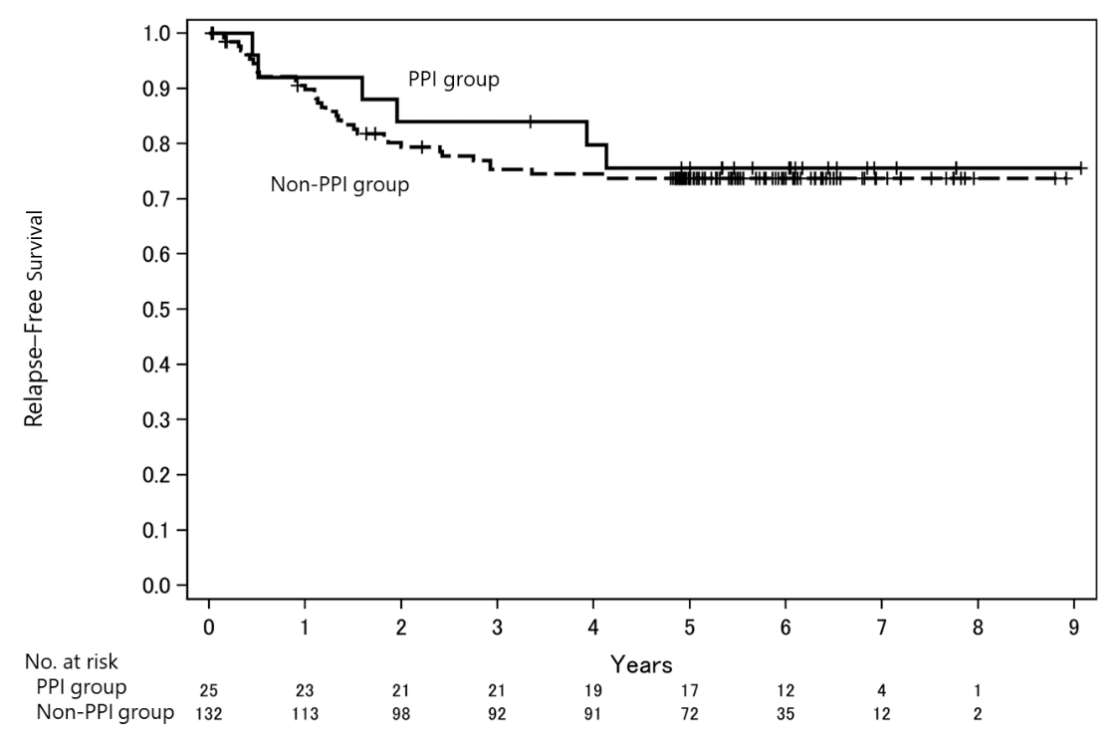


**b**


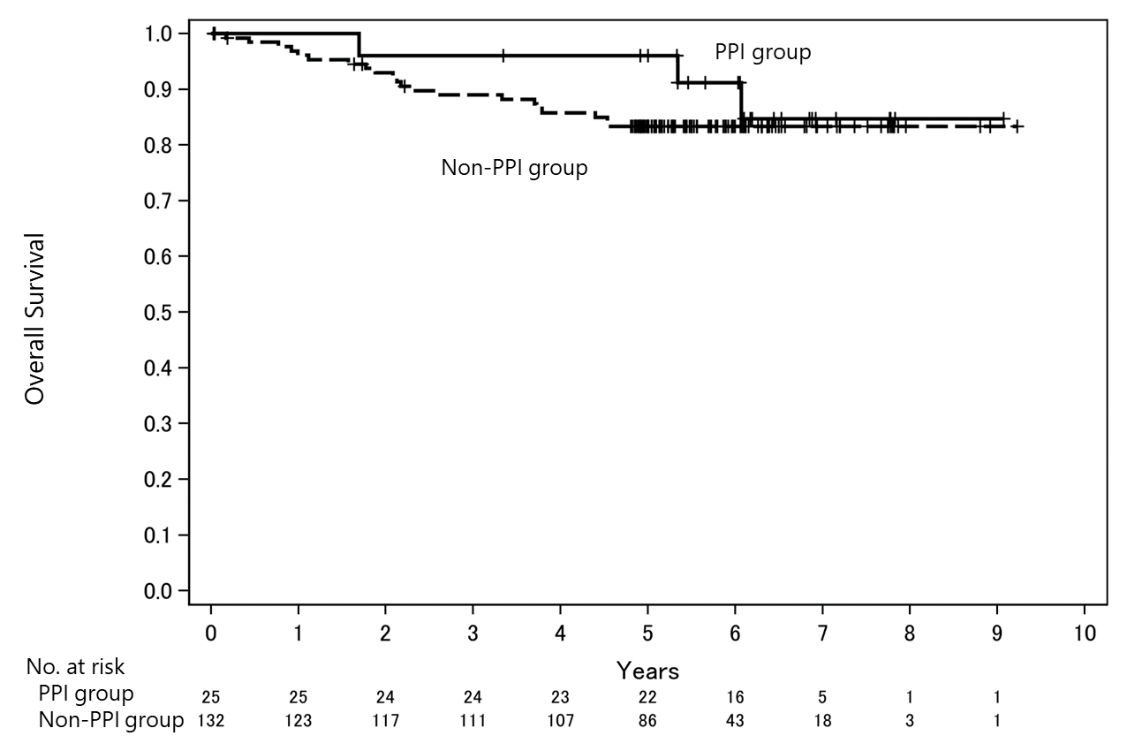


**Supplementary Figure S2.** Kaplan–Meier curves for (**a**) relapse-free survival and (**b**) overall survival according to the absence or presence of PPIs for the CapeOX regimen. Abbreviations: PPI, proton pump inhibitor.
